# Safety of wireless capsule endoscopy in patients with implantable cardiac devices

**DOI:** 10.1002/jgh3.12251

**Published:** 2019-09-04

**Authors:** Christopher Kasia, Anoop Appannagari, Anjali Joshi, Mukund Venu

**Affiliations:** ^1^ Department of Internal Medicine Loyola University Medical Center Maywood Illinois USA; ^2^ Division of Gastroenterology and Nutrition Loyola University Medical Center Maywood Illinois USA; ^3^ Division of Cardiology Loyola University Medical Center Maywood Illinois USA

**Keywords:** gastrointestinal bleeding, internal cardiac defibrillator, left‐ventricular assist device, small bowel, wireless capsule endoscopy

## Abstract

**Background and Aim:**

Wireless capsule endoscopy (WCE) has become an increasingly utilized imaging modality for the evaluation of gastrointestinal bleeding. There is a paucity of data evaluating the safety and use of WCE in patients with implantable cardiac devices.

**Methods:**

A retrospective chart review of all patients who had a WCE at Loyola University Medical Center in Maywood, IL, USA completed between January 2007 and December 2016 identified patients with internal cardiac devices and obscure gastrointestinal bleeding. Patient WCE footage was viewed in its entirety before creating a final report to ensure no gaps in footage and video quality.

**Results:**

No patient complaints were documented during the 8‐h procedure duration, and there were no cardiac abnormalities noted on telemetry. There were no device‐related complications documented in the 30‐day postprocedure time period. Postprocedure analysis of the WCE recordings demonstrated no interference in WCE image quality (loss of images or gaps in video) or duration.

**Conclusions:**

There is no significant interference between WCE and implantable cardiac devices, and it appears to be safe to use.

## Graphical abstract

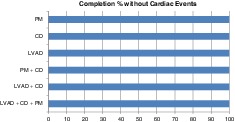

Our study showed no adverse cardiac outcomes in patients with implantable cardiac devices receiving capsule endoscopy. Data quality from capsule endoscopy is not compromised in patients with implantable cardiac devices.

## Introduction

Wireless capsule endoscopy (WCE) has become an increasingly utilized imaging modality for suspected small bowel bleeding or occult obscure gastrointestinal bleeding and inflammatory bowel disease.^1^ It has been shown to have a diagnostic yield close to 60–70% and is helpful in locating a bleeding source undetected by other modalities.^2^

Recently, WCE has becoming increasingly used in patients with cardiac devices such as pacemakers, internal cardiac defibrillators (ICD), and left‐ventricular assist devices (LVAD).^3^ However, manufacturers have proposed a theoretical electromagnetic interference between the capsule and the cardiac device itself.^4^, ^5^ The origins of this theory arose from observations in patients with implantable cardiac devices who underwent radiofrequency ablation and were noted to have device malfunction and failure during such an intervention.^6^ Such observations have led the U.S. Food and Drug Administration (FDA) to adopt a relative contraindication to the use of WCE in patients with implantable cardiac devices.^7^

There is a paucity of data detailing the safety and use of WCE in patients with implantable cardiac devices.^8^, ^9^ The use of some of these cardiac devices, most notably LVADs, increases the risks of gastrointestinal bleeding, with as much as 20–40% of LVAD recipients developing gastrointestinal bleeds.^10^ With up to 65% of these bleeds coming from an unidentified source after completing upper and lower endoscopies, use of WCE is increasingly relevant in these patients.^11^, ^12^

There is no consensus on how to best monitor the true short‐ and long‐term safety of this procedure and subsequent patient cardiac safety events. There are small retrospective studies that have evaluated the use of WCE in patients with cardiac devices, specifically focusing on the safety of such devices with regard to cardiac events.^12^, ^13^, ^14^ Although the initial data are promising, they comprise small case studies of only a handful of patients or are only focused on a specific cardiac device.^7^, ^15^, ^16^

The primary aim of this study was to investigate adverse cardiac outcomes in patients with any implantable cardiac devices who had undergone WCE. Secondary outcomes of this study included an evaluation of the quality of the data obtained through WCE to see if cardiac devices interfere with the images produced.

## Methods

A retrospective chart review of all patients who had a WCE at Loyola University Medical Center (LUMC) in Maywood, IL, USA completed between January 2007 and December 2016 was performed to identify patients with internal cardiac devices. Inclusion criteria were patients aged 18 and older with obscure gastrointestinal (GI) bleeding—hematemesis, melena, hematochezia, or anemia with positive fecal occult blood test; who had documentation of having an implanted cardiac device—left ventricular assist device, ICD, or pacemaker; who had undergone a WCE; and who were monitored in the LUMC GI lab. Excluded from this study were patients younger than 18 years of age. The Loyola University Institutional Review Board approved this study prior to initiation.

Patient records were reviewed for demographic data, indication for WCE, and integrity of the quality of the data captured. Charts were reviewed for potential cardiac complications during the WCE up to 30 days postprocedure.

Patients received a brochure detailing proper preparation ahead of a WCE. Patients were instructed to initiate a clear liquid diet the afternoon prior to the procedure and complete standard bowel preparation the night prior with 1 gallon of Golytely (Braintree Laboratories, Braintree, MA, USA). Patients were required to be nil per os (NPO) at midnight the night prior to the procedure. They were allowed to continue to take their oral medications, including all cardiac medications.

The capsules used for the WCE were the PillCam SB2 and SB3 systems (Medtronic [formerly Given Imaging], Duluth, GA, USA). Results were interpreted using associated software by experienced physicians. Patients were monitored on continuous telemetry during the entire WCE procedure and were evaluated serially by the GI staff for any cardiac, respiratory, or abdominal complaints. LVADs were continuously monitored by their associated system controller. No prokinetic medications were used. After 2 h, a clear liquid diet was allowed, followed by a light lunch after 4 h. After 8 h, the recorder and sensor belt were removed, and the WCE was completed. Each patient's capsule endoscopy footage was reviewed in its entirety before creating the final report. Quality of WCE was assessed by loss of images or gaps in video duration.

### *Statistical analysis*

Patient characteristics and clinical data are presented as means, counts, and percentages for categorical variables. Tables denoting patient demographics, indications for WCE, device characteristics, and patient device combinations are listed in numerical order.

## Results

A total of 112 WCE procedures were performed in 83 patients with implantable cardiac devices over a 9‐year time period. In 20 patients, the WCE was repeated multiple times due to a new diagnosis of anemia, abdominal pain, or GI bleed. Of the 83 patients, 53 (64%) were male, and 30 (36%) were female. The average patient age was 63 years, with an age range of 23–94 years. Patient demographics and indications for WCE can be found in Table 1.

**Table 1:** Demographic data and indications for study

Patient demographics	*n* (%)
Number of capsule studies	112
Number of patients	83
Gender	
Male	69 (67)
Female	43 (33)
Average age	68 y/o (range 23–94)
Indications for capsule study	
Abdominal pain	5 (4.5)
Anemia	72 (64.3)
Crohn's disease	1 (0.9)
Gastrointestinal bleeding	28 (25)
Melena	10 (8.9)

The majority of WCE studies were performed for anemia (64%) or GI bleed (25%). Other indications included melena (8.9%), abdominal pain (4.5%), and Crohn's disease (0.9%). The inclusion of melena as its own category, separate from GI bleed, was carried out to best express the clinical presentation of the patients who received WCE. The melena group included patients whose reason for WCE was active melenic stools, while those in the GI bleed group presented with GI‐related blood loss other than melena.

The majority of patients had a single permanent pacemaker (44%), followed by patients with LVAD and ICD (17%). Of patients who underwent WCE, 25% had an LVAD. Patient device combinations can be found in Table 2 and distribution of devices by manufacturer in Table 3.

**Table 2:** Implantable cardiac device information and device‐related event‐monitoring results

Type of ICD		*n* (%)	
PM		49 (44)
CD		18 (16)
LVAD		3 (2.6)
PM + CD		17 (15)
LVAD + CD		19 (17)
LVAD + CD + PM		6 (5.4)

Device‐related event‐monitoring results		
	Intraprocedure	30 days Postprocedure
Changes during study	None	None
Inappropriate ATP	None	None
Inappropriate shocks	None	None
Inappropriate sensing	None	None
Cardiac arrhythmias	None	None
Patient complaints	None	None

ATP, antitachycardia pacing; CD, cardiac defibrillator; ICD, implantable cardiac device; LVAD, left ventricular assist device; PM, pacemaker.

**Table 3:** Manufacturer and implantable cardiac device type

Manufacturer	PM	CD	PM + CD	LVAD
Medtronic	23	10	12	0
Boston Scientific	13	4	4	0
Biotronik	0	2	0	0
St. Jude Medical	8	2	1	0
Unknown	5	0	0	0
Heartmate II	0	0	0	28
Heartware	0	0	0	2

CD, cardiac defibrillator; LVAD, left ventricular assist device; PM, pacemaker.

No patient complaints were documented during the 8‐h procedure duration, and there were no cardiac abnormalities noted on telemetry. In the patient chart review, there were no device‐related complications documented in the 30‐day postprocedure time period.

Postprocedure analysis of the WCE recordings demonstrated no interference in WCE image quality (loss of images or gaps in video) or duration.

## Discussion

The use of WCE for small bowel obscure or occult gastrointestinal bleeding has been increasing recently. Most capsule manufacturers list wireless capsules as a contraindication in patients with implantable cardiac devices because of possible interference between the electromagnetic signal transmitted by the capsule and the cardiac device itself.^4^ Our aim in this study was to investigate if patients with these devices, who had undergone a WCE procedure, had any adverse cardiac outcomes. Our main findings were that there was no cardiac interference or abnormalities noted during the 8‐h WCE procedure or during the 30‐day postprocedure time period. We also evaluated the quality of data obtained during the WCE. Cardiac devices did not affect the quality of the images that were produced during the procedure.

Our findings are consistent with studies that show there is little to no effect on implantable cardiac devices due to any electromagnetic interference by the wireless capsule. There are studies which have cautioned that wireless telemetry may impair recording of images transmitted by the capsule.^1^, ^4^ However, our study did not demonstrate any instances where this was the case.

Irrespective of the number of devices a patient has, the device sizes, or the device manufacturer, no patient suffered any impact on their cardiac device, and their footage quality remained unaltered.

There are a few important study limitations to highlight. In our evaluation of the WCE effect on footage quality, as well as device and cardiac safety, we did not evaluate capsule study completeness. We also did not focus on whether the actual bleeding source was identified or not. With our focus on patient safety, the diagnostic outcome was not evaluated or reported. The actual capsules used in our study were not uniform. Two distinct models were used. It is possible that the technological advancements between the capsule models were able to impact the cardiac safety and footage quality outcomes. However, we do not suspect this to be an issue given that, despite both models used, there were no adverse cardiac outcomes or decrease in footage quality noted.

There has been concern about the usage of WCE in patients who have implantable cardiac devices. With an increasing use of implantable cardiac devices to sustain an aging population with multiple complex comorbidities, the use of WCE is going to continue to increase.^17^ The elderly, and especially those with implantable cardiac devices, are more prone to gastrointestinal bleeds. This is particularly true in those with LVADs who require lifelong anticoagulation due to the unique vascular flow produced by the device itself. Being able to use a simple and minimally invasive method of investigation is of benefit to the patient and to the provider. It is safer and has less procedural risk than an endoscopy. As we have demonstrated, the capsule itself is well tolerated by such patients from a cardiac perspective. The results are not obscured by the presence of an implantable cardiac device. It is important to lift or modify the FDA warnings on the use of WCE in this patient population. The device type, number of devices, combination of devices, and specific manufacturer did not impact the results of our study. Thus, there is no need to adjust industry standards for such device manufacturing if they have all been shown to be safe from a cardiac perspective. Multiple case studies show that there is no loss of data quality or cardiac device compromise. To our knowledge, the LVAD population included in our work is the largest of its kind to have been evaluated in such a study, with 25% of our patient population having an LVAD alone or in combination with other implantable cardiac devices. Due to the evolution of advanced heart failure therapies in the setting of an aging patient population with increasingly complex medical comorbidities, it will be much more common to encounter such patient and clinical scenarios, especially because these patients require lifelong anticoagulation due to the nature of their device. Based on the results of our study and corroborating literature on the topic, there is no significant interference between WCE and implantable cardiac devices, and WCE appears to be safe to use.
